# *CYP17A1* Intron Mutation Causing Cryptic Splicing in 17α-Hydroxylase Deficiency

**DOI:** 10.1371/journal.pone.0025492

**Published:** 2011-09-26

**Authors:** Daw-Yang Hwang, Chi-Chih Hung, Felix G. Riepe, Richard J. Auchus, Alexandra E. Kulle, Paul-Martin Holterhus, Mei-Chyn Chao, Mei-Chuan Kuo, Shang-Jyh Hwang, Hung-Chun Chen

**Affiliations:** 1 Division of Nephrology, Department of Medicine, Kaohsiung Medical University Hospital, Kaohsiung, Taiwan; 2 Division of Pediatric Endocrinology, Department of Pediatrics, Christian Albrechts University, Kiel, Germany; 3 Division of Metabolism, Endocrinology and Diabetes, Department of Internal Medicine, University of Michigan Medical School, Ann Arbor, Michigan, United States of America; 4 Division of Genetics, Endocrinology and Metabolism, Department of Pediatrics, Kaohsiung Medical University Hospital, Kaohsiung, Taiwan; 5 Department of Medical Genetics, College of Medicine, Kaohsiung Medical University, Kaohsiung, Taiwan; 6 Faculty of Renal Care, College of Medicine, Kaohsiung Medical University, Kaohsiung, Taiwan; Universitat de Barcelona, Spain

## Abstract

17α-hydroxylase/17, 20-lyase deficiency (17OHD) is an autosomal recessive disease causing congenital adrenal hyperplasia and a rare cause of hypertension with hypokalemia. The *CYP17A1* gene mutation leads to 17OHD and its clinical features. We described an 18 y/o female with clinical features of 17α-hydroxylase/17, 20-lyase deficiency and characterized the functional consequences of an intronic *CYP17A1* mutation. The coding regions and flanking intronic bases of the *CYP17A1* gene were amplified by PCR and sequenced. The patient is a compound heterozygote for the previously described p.R358X and IVS1 +2T>C mutations. A first intron splice donor site mutation was re-created in minigene and full-length expression vectors. Pre-mRNA splicing of the variant *CYP17A1* intron was studied in transfected cells and in a transformed lymphoblastoid cell line. When the full-length *CYP17A1* gene and minigene containing the intronic mutation was expressed in transfected cells, the majority (>90%) of mRNA transcripts were incorrectly spliced. Only the p.R358X transcript was detected in the EBV-transformed lymphoblastoid cell line. The IVS1 +2T>C mutation abolished most 17α-hydroxylase/17, 20-lyase enzyme activity by aberrant mRNA splicing to an intronic pseudo-exon, causing a frame shift and early termination.

## Introduction

17α-hydroxylase deficiency (17OHD), a rare autosomal recessive disease caused by *CYP17A1* gene mutation, accounts for about 1% of all congenital adrenal hyperplasia and is characterized by hypertension, hypokalemia, and sexual infantilism [1]. The *CYP17A1* enzyme catalyzes two different enzymatic reactions: 17α-hydroxylation of progesterone and pregnenolone and the 17,20 lyase reaction for the conversion of 17-hydroxypregnenolone to dehydroepiandrosterone (DHEA). Defects in these two enzymatic reactions lead to decreased glucocorticoid and sex steroid production with mineralocorticoid excess, primarily 11-deoxycorticosterone and corticosterone, as well as 18-hydroxydeoxycorticosterone and 18-hydroxycorticosterone. Clinically, patients present with lack of pubertal development, hypertension, and hypokalemia with renal potassium wasting [2].

There are more than 70 inactivation mutations of *CYP17A1* leading to 17OHD being reported, and six of them are intron mutations [3]–[8]. Four intron mutations showed “exon skipping” to the next intron-exon boundary or to cryptic adjacent splice sites by *in vitro* analysis using partial or full-length *CYP17A1* gene, whereas no functional studies were performed on the two first intron mutations. In the present study, we describe a patient with a first intron splice donor site mutation and characterize the aberrant splicing in transfected cells and in lymphoblastoid cells derived from the patient.

## Materials and Methods

### Ethics Statement

The study protocol was approved by the institutional review board of the Kaohsiung Medical University Hospital (KMUH-IRB-980005). Informed consents have been obtained in written form from patient and her relatives and all clinical investigation was conducted according to the principles expressed in the Declaration of Helsinki. The patient and her relatives gave consent for the publication of the clinical details.

### Case Report

A Chinese female of non-consanguineous parents was evaluated due to irregular menstruation, no breast development, and no axillary and pubic hair at the age of fourteen. Ovarian failure and hypogonadism was diagnosed, and she received estradiol and progesterone treatment since then. Four years later, at the age of 18, she visited our facility for further evaluation. Chromosome study showed normal 46,XX karyotype. Pelvic ultrasound showed retarded uterus development (7.63 cm×1.47 cm×3.69 cm) and bilateral ovaries (right: 2.33 cm×0.91 cm×2.45 cm and left: 2.24 cm×0.78 cm×2.49 cm). The breast showed Tanner stage 3 development. The bone age was significantly retarded at 13 years, and her height was 170 cm (height-for-age z-score = +2.33). She was referred to nephrology due to hypertension, hypokalemia, and mild proteinuria. At initial visit, her blood pressure was 160/100 mmHg, potassium level of 3.1 mEq/liter, urine protein/creatinine ratio of 0.54 (mg/mg), and transtubular potassium gradient (TTKG) of 10.49. Plasma renin, aldosterone, and other biochemistry data are listed in Table 1. Mineralocorticoids and glucocorticoids (Table 1) were measured by ultrapressure liquid chromatography tandem mass spectrometry (UPLC-MS/MS) according to the previously described method [9]. In brief, aliquots of samples, calibrators and controls were extracted by solid phase extraction (SPE) using Oasis MAX SPE system plates (Waters, Milford, MA, USA). Deuterium labeled steroids were used as internal standards. The UPLC-MS/MS system was used in the multiples reaction monitoring mode (MRM) and steroids were measured in the positive ion mode except aldosterone which was measured in the negative mode. For each hormone two different MRM transitions were monitored. The limit of quantification was between 0.1 nmol/L for 17-hydroxyprogesterone and 2 nmol/L for cortisol. The intra- and inter assay coefficient of variations (%CV) for replicate quality controls for different concentrations range between 2.4% and 9.7%. Total run time for the assay was 5 min. Clinically, 17α-hydroxylase deficiency was suspected. After 2 months of 10 mg prednisolone daily, her blood pressure improved with disappearance of hypokalemia and proteinuria.

**Table 1:** Patient's adrenal steroid metabolites.

Hormones	Patient	Reference ranges
ACTH, pg/ml (pmol/l)	272 (59.84)	<60 (13.2)
Estradiol, pg/ml (pmol/l)	10.9 (<40.3)	60–200 (222–740)
DHEA Sulphate, µg/dl (µmol/l)	3.7 (0.0987)	130–980 (3.47–26.13)
Progesterone*, ng/ml (nmol/l)	1.8 (5.72)	0.03–1.84 (0.095–5.85)
11-Deoxycorticosterone*, ng/ml (nmol/l)	2.9 (8.75)	0.03–0.36 (0.09–1.09)
Corticosterone*, ng/ml (nmol/l)	71.9 (207.5)	0.28–4.59 (0.808–13.25)
Aldosterone, pg/ml (nmol/l)	205.2 (56.94)	40–310 (11.1–86.03)
17-OH progesterone*, ng/ml (nmol/l)	<0.03 (0.091)	0.14–0.79 (0.426–2.406)
11-Deoxycortisol*, ng/ml (nmol/l)	<0.03 (0.087)	0.03–2.2 (0.087–6.36)
Cortisol*, ng/ml (nmol/l)	14 (38.6)	50–249 (137.9–686.8)
Cortisone*, ng/ml (nmol/l)	<0.03 (0.083)	3.7–28.0 (10.26–77.68)
Androstenedione*, ng/dl (nmol/l)	<3 (104.9)	3–157 (0.105–5.49)
Testosterone*, ng/dl (nmol/l)	<5 (<0.1735)	3–49 (0.1041–1.7)
Renin, pg/ml (nmol/l)	<2.55 (60.4)	5.1–38.7 (120.9–917.2)

ACTH, Adrenocorticotropic hormone; DHEA, dehydroepiandrosterone.

SI units were given in parentheses. Asterisk (*) indicates analytes assayed by LC-MS/MS.

### DNA, RNA Isolation and Sequencing

Genomic DNA was extracted from peripheral blood leukocytes of the patient, her mother, her brother, and a maternal uncle. The eight exons and intron-exon boundaries of *CYP17A1* gene were amplified by PCR using primers and conditions described previously [10]. PCR products were confirmed by electrophoresis in 1.5% agarose gel and sequenced by ABI 3730XL DNA Analyzer (Applied Biosystems, Foster City, CA). Total RNA was collected from the patient's peripheral blood leucocytes by the standard Trizol method (Invitrogen, Carlsbad, CA). The reference sequences of *CYP17A1* gene and mRNA were NG_007955.1 and NM_000102.3, respectively.

### Minigene and Full-Length Gene Construction

The genomic *CYP17A1* in pcDNA3 (Invitrogen, Carlsbad, CA) vector was used as previously described [7]. A wild type *CYP17A1* minigene, which spans exon 1 to exon 3, was made by cutting the whole *CYP17A1* vector with EcoRI and self-ligation of the resulting plasmid. The IVS1 +2T>C minigene mutant was created by QuikChange Mutagenesis kit (Stratagene, Cedar Creek, Texas, USA) with primer pair 5′-GCGGCCTCAAATGGCAAGTGGTGCCCATC-3′ and 5′- GATGGGCACCACTTG CCATTTGAGGCCGC-3′, while the full-length mutant was made with 5′-CTGGGCGGCCTCAAATGGCAAGTGGTGCCCATCTCCTCCC-3′ using a single primer method [11], and verified by partial sequencing.

### Transient Transfection, RT-PCR, and Western Blot

HEK-293 cells and COS-7 cells were obtained from American Type Culture Collection (Manassas, VA). Cells were transfected with equal amount of plasmids containing the wild-type or mutated *CYP17A1* minigene by FuGENE6 (Roche, USA) according to the manufacture's protocol. Cells were harvested 24 h after transfection and total RNA or total protein was extracted followed by RT-PCR and Western blot analysis [12]. The RNA samples were reverse transcribed with the M-MLV RT-PCR kit (Promega, USA). Primers a-e used for PCR are: a, 5′-GCCACCATGTGGGAGCTCGTG-3′; b, 5′-TGGCTCTCTTGCTGCTTACC-3′; c, 5′-GTTGTTGGACGCGATGTCTA-3′; d, 5′-GGGGACTAGGTCCACCAGGCT-3′; e, 5′-TGTGATGCAGCGCCCACAGA3′. The RT-PCR products were separated on 1.5% agarose gels, purified, and directly sequenced or subcloned by the TOPO TA Cloning Kit (Invitrogen, Carlsbad, CA, USA) when needed. Lymphoblastoid cell lines (LCL) were established by EBV-transformation of lymphocytes from the patient as described [13]. Goat anti-CYP17A1 polyclonal IgG (N-18, sc-46085, Santa Cruz Biotechnology) was used in the Western blot analysis.

## Results

**Figure 1:**
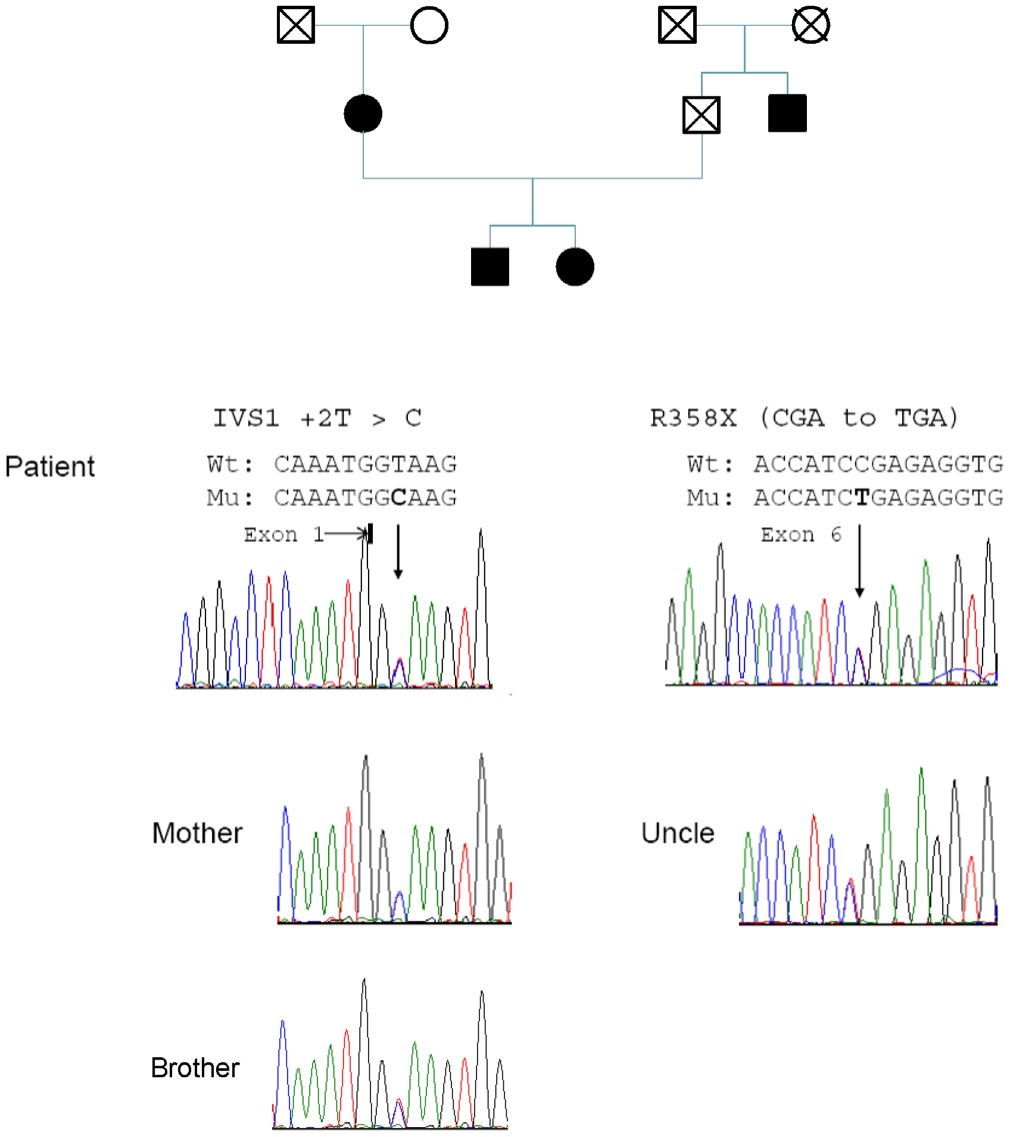
Pedigree analysis of *CYP17A1* gene. The patient is found to be a compound heterozygous for mutations of *CYP17A1*. The first mutation was identified as IVS1 +2T>C and the second mutation p.R358X (CGA→TGA). The first intron mutation is originated from her mother and p.R358X from her father, based on the analysis of her uncle's genomic DNA. Proband's brother is also heterozygous for the affected maternal allele.

Sequencing of the eight exons and intron-exon boundaries of *CYP17A1* gene showed compound heterozygous mutations at position IVS1 +2T>C in intron 1 and p.R358X in exon 6. Analysis of DNA from her mother and paternal uncle showed that the p.R358X mutation derived from the father while mutation IVS1 +2T>C was inherited from her mother (Fig. 1). The IVS1 +2 T>C and R358X mutations both have been reported previously [8], [14].

**Figure 2:**
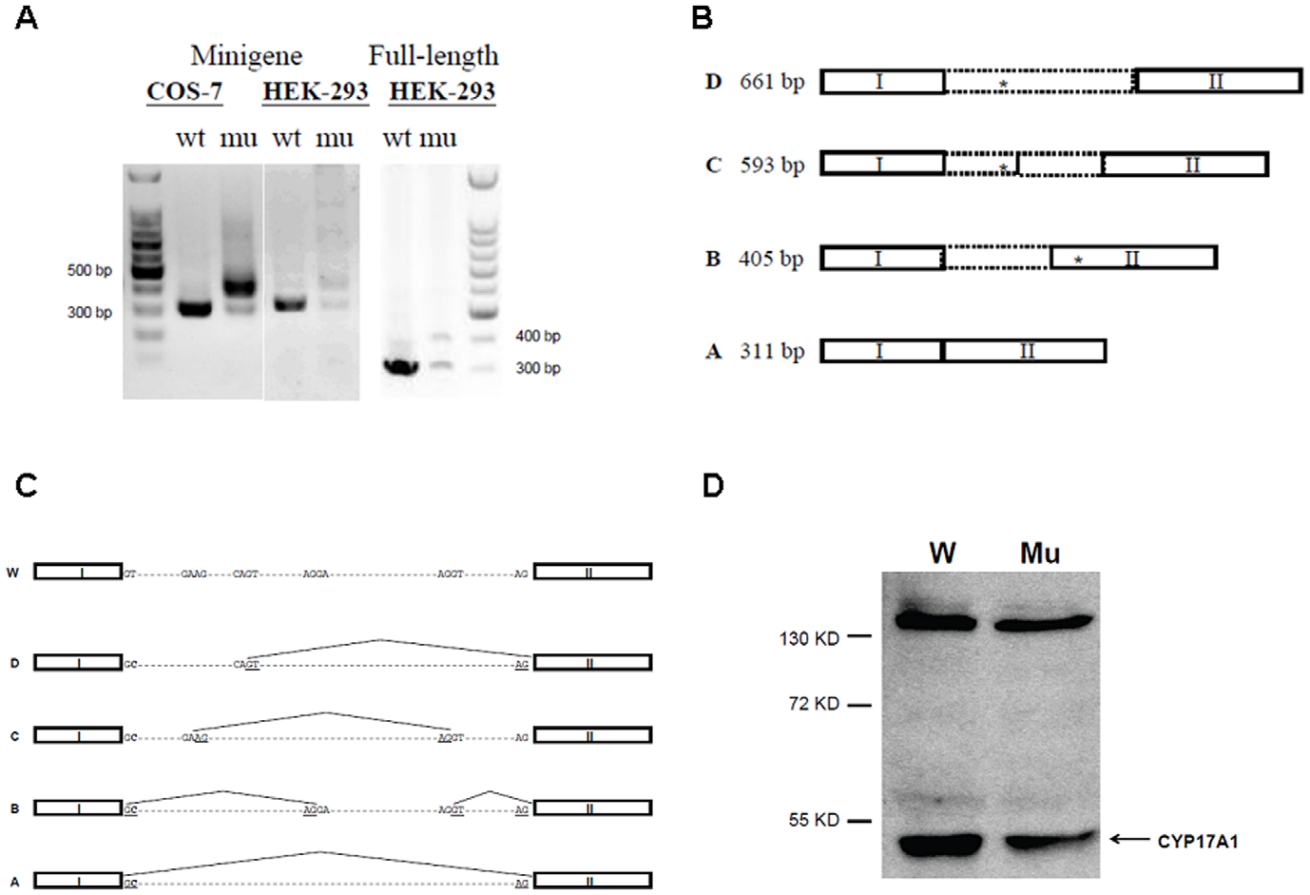
Heterologous expression and mRNA analysis using minigene and full-length *CYP17A1* gene, wild-type and mutation IVS1 +2T>C. A) RT-PCR analysis shows only the correct 311 bp amplicon when wild-type *CYP17A1* minigene or full-length gene is expressed in COS-7 or HEK293 cells. In contrast, the IVS1 +2T>C mutant *CYP17A1* gene affords primarily a ∼400 bp amplicon, best demonstrated in COS-7 cells, as well as lesser amounts of the normal 311 bp amplicon and larger molecular species. B) Cartoon demonstrating size and sequence of aberrantly spliced transcripts obtained from transfected HEK293 cells expressing the IVS1 +2T>C *CYP17A1* mutation, obtained after TOPO TA subcloning and sequencing. The asterisks indicate position of in-frame termination codons. C) Cartoon demonstrating splice sites used to generate transcripts obtained from transfected HEK293 cells expressing the IVS1 +2T>C *CYP17A1* mutation. Figure length is not drawn to scale. D) Western blot analysis of protein obtained from transiently transfected HEK293 cells expressing the wild type (W) and IVS1 +2T>C mutation (Mu) full-length *CYP17A1* gene. Decreased CYP17A1 protein expression was noted for the IVS1 +2T>C mutant full-length *CYP17A1* gene compared to the wild-type gene.

To analyze the effect of first intron donor site mutation on the *CYP17A1* mRNA splicing, *CYP17A1* minigenes were constructed (Fig. 2A) and transiently transfected into HEK-293 and COS-7 cells. The wild type minigene expressed only the expected 311 bp mRNA product in both COS-7 and HEK-293 cells (Fig. 2B). In contrast, the minigene construct containing the IVS1 +2T>C mutation primarily yielded a ∼400 bp RT-PCR product, plus a trace of the 311 bp transcript (Fig. 2A). After TOPO cloning and sequencing, a total of 4 different mRNA products, A to D, were identified (Fig 2B). A 1983 bp fragment corresponding to the genomic DNA sequence derived from either residual DNA or unspliced pre-mRNA.

Analysis of these four mRNA variants from HEK-293 cells revealed different splicing sites for each transcript. Variant A was identical to the wild type sequence with length of 311 bp, indicating a fraction of proper pre-mRNA splicing despite the mutation. In variants B to D, pseudo-exons of various lengths were created within the first intron using different cryptic splicing donor and acceptor sites (Fig. 2C). A downstream stop codon was generated in these three alternatively spliced mRNA forms, either within the original intron region (variant C and D) or in the second exon due to frame shifting (variant B). Immunoblots of protein from cells expressing the mutant *CYP17A1* gene contained reduced amounts of CYP17A1 protein compared to cells expressing the wild-type gene (Fig 2D).

**Figure 3:**
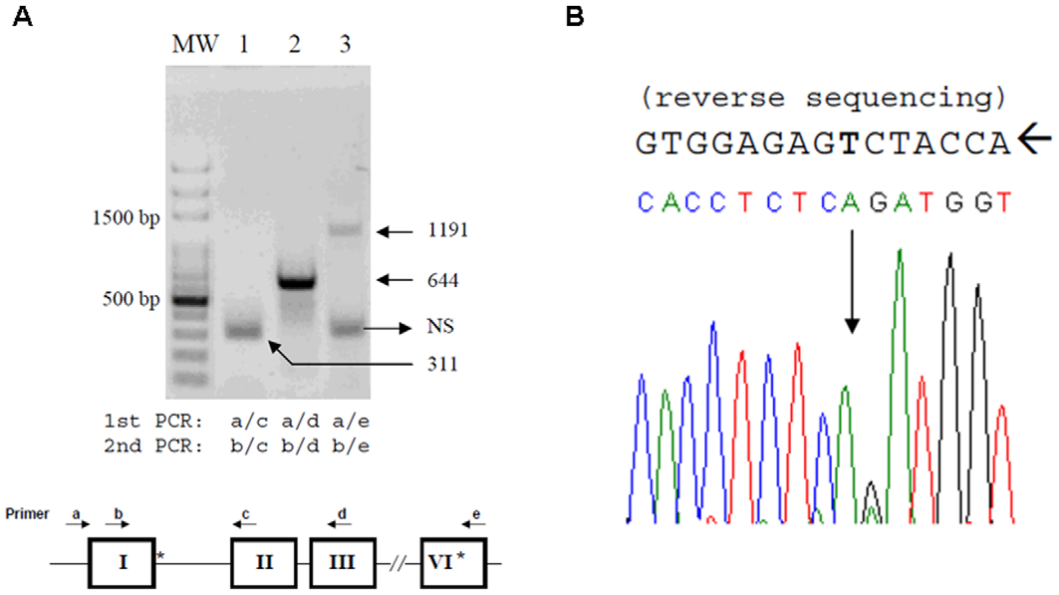
RT and semi-nested PCR analysis of *CYP17A1* mRNA from the patient's LCL. A) Amplicons were derived from RT-PCR spanning exons 1 and 2 (lane 1, 311 bp band), exons 1 and 3 (lane 2, 644 bp band), or exons 1 and 6 (lane 3, 1191 bp band). The PCR primers used are indicated at bottom of the figure panel; N.S. indicates a non-specific PCR product. The 1191bp PCR product spans from exon 1 to exon 6, with primer e designed just 3′ to the p.R358X position. B) Direct sequencing (reverse strand) analysis of the 1191bp band showed only the p.R358X mutation without wild type sequence.

While a single round of RT-PCR yield no visible bands or irreproducible results from RNA isolated from patient's peripheral blood and LCL (data not shown), semi-nested PCRs performed on RNA from the patient's LCL gave a predominant ∼300 bp-amplicon using a primers in the first and second exons (primers b and c, Fig 3A, lane 1). After TA cloning of the PCR products, two out of twenty clones were ∼400 bp amplicons, and sequencing identified this fragment to be the same as the major form B obtained from transfected cells. RT-PCR using primers in exons 1 and 3 or exons 1 and 6 (primers b and d or primers b and e) yielded the expected amplicons of 644 and 1191 bp. Direct sequencing of the 1191 bp fragment showed only the paternal p.R358X sequence (Fig 3B), suggesting that most if not all full-length *CYP17A1* mRNA in the LCL derives from the p.R358X allele.

## Discussion

The *CYP17A1* p.R358X mutation has been reported in one girl of Korean descent with combined 17α-hydroxylase/17,20-lyase deficiency, who presented with primary amenorrhea, sexual infantilism, and hypertension [14]. Although p.R358Q caused isolated 17,20-lyase deficiency [15], the p.R358X mutation produces a premature stop codon and encodes a protein lacking the heme moiety essential for catalytic activity. The IVS1 +2T>C splicing donor site mutation was reported to database without clinical description and functional characterization [8]. We find that over 90% of the pre-mRNA is aberrantly spliced, eliminating most but not all enzymatic activity and explaining the origin of low but detectable serum cortisol and breast development in this severely affected patient. Presumably, non-sense mediated degradation further reduced the abundance of mRNA transcripts from both mutant alleles relative to wild-type *CYP17A1* (Figs 2 & 3). The suppression of aldosterone in 17OHD is variable [1], even within the same family [16]. Normal or even high aldosterone with suppressed renin has been reported [4], [10], [16]–[18]. The factors leading to detectable aldosterone in 17OHD are not known [19]. In contrast, 18-hydroxydeoxycorticosterone and 18-hydroxycorticosterone are often elevated in 17OHD [20], due to elevated precursor synthesis and the weak 18-hydroxylase activity of CYP11B1 in the zona fasciculata. Because 18-hydroxycorticosterone is the immediate precursor to aldosterone, aldosterone production in some 17OHD cases might reflect either synthesis driven by precursor excess or cross-reactivity in the clinical assays. Furthermore, hypertension is not caused by aldosterone excess in 17OHD, thus the variability in hypertension is not caused by variable aldosterone concentrations.

Ideally, direct demonstration of the functional consequences of *CYP17A1* intronic mutations requires analysis of either gonadal or adrenal tissue to confirm the finding in model systems [5], [21]. Inconsistent RT-PCR analysis was found between RNA from peripheral blood cell and tissue in other cases of 17OHD, which might be due to the degradation of large hnRNA in the tissue [21]. This approach is problematic since many 17OHD patients do not need surgical removal of their gonad and adrenal glands, except for cryptorchid testes in patients with a 46,XY karyotype due to potential malignancy. Alternatively, the use of heterologous minigene expression studies, although somewhat artificial, usually predicts cryptic splicing sites with high accuracy. Previous use of full-length *CYP17A1* gene expression demonstrated the feasibility of simultaneous protein and RNA analysis to characterizing intronic mutations [7].

Accurate pre-mRNA splicing is catalyzed by the spliceosome, a large complex composed of five small nuclear ribonucleoproteins (snRNPs), which recognizes conserved sequences at the exon-intron boundaries and branch point sites [22]. The 5′ splicing site (5′ss) is composed of nine partially conserved nucleotides at the exon-intron boundary, and base pairing to the U1 snRNA 5′ terminus occurs in this complex [23]. The pre-mRNA introns can be divided into U2 and U12 type depending on the spliceosome being used. The majority of mammalian introns are U2 type and consist of three subtypes: GT-AG, GC-AG and AT-AC [24]. Previously observation showed that mutation at 5′ss position +2 can be tolerated but with decreasing order in splicing efficiency as +2T > +2C > +2A > +2G [25], [26]. This 5′ss +2 site mutation is intrinsically weak because of a mismatch base pair with U1 snRNA, although the identity of hydrophobicity coefficients of T and C may compensate for the mismatch at position +2 of GC-AG introns [27]. These GC-AG splicing sites account for 0.56%–1.28% of splice donor sites and may exist in one in every 20 alternative mRNA isoforms [28]–[30].

By using vectors for expressing a *CYP17A1* minigene and full gene, we found the mRNA splicing products are similar, but the minigene expression vector produced other cryptic mRNAs. This result may indicate the existence of cis-elements regulating the first intron splicing in the downstream region of the minigene. The IVS1 +2T>C mutation utilized the non-canonical GC-AG splicing donor site, albeit inefficiently, as suggested by the immunoblot result (Fig 2D). If this GC-AG site was used *in vivo*, although low in abundance, then normal *CYP17A1* mRNA transcript and protein would be generated, reducing the severity of the phenotype. In the patient's LCL, we detected only *CYP17A1* mRNA spanning exons 1-6 derived from the paternal p.R358X allele, yet we cannot exclude a small fraction of wild-type *CYP17A1* transcript using direct sequencing studies. Furthermore, the patient clinically has severe 17OHD deficiency, with negligible 19-carbon steroid production yet detectable cortisol and partial breast development. It is difficult to predict how well the non-canonical GC-AG splicing donor is tolerated in the human gonad and adrenal gland based on data obtained from heterologous expression systems or from ectopic *CYP17A1* expression in the LCL. Our data suggest that a trace of wild-type mRNA might be produced, which indicates that the use of non-canonical splicing sites *in vivo* might be under-estimated because of their low expression level. The determinants of non-canonical GC-AG splicing donor usage *in vivo* are unclear, and the loss of trans-acting factors regulating splicing in the heterologous expression systems may contribute to these differences. Discrepancies in minigene experiments, which failed to fully recapitulate the normal mRNA splicing pattern, was also reported in studies of a splicing site mutation of the calcium sensing receptor [31].

In summary, we identified a *CYP17A1* first intron splicing site mutation in heterozygous combination with p.R358X leading to the severe 17OHD. Discrepancies between transient transfection and LCL experiments were found, highlighting the limitations of using such model systems for the study of mRNA splicing in the adrenal and gonad. Based on the clinical and experimental data, it is likely that the *CYP17A1* IVS1 +2T>C severely but not completely disrupts pre-mRNA splicing. In the analysis of intronic mutations, a combined use of minigene, full-length gene, LCL, and native tissues may be necessary to accurately determine the functional changes caused by the mutation.
